# An Obscure Case

**Published:** 1877-07

**Authors:** 


### AN OBSCURE CASE.

A professional brother presents the following case, and asks for information and advice.

"The patient is a lady about thirty-seven years of age: general health good: all her teeth are in good condition, except the left superior central incisor. This tooth is perfectly sound, so far as decay is concerned: their is no tarter, no recession of the gum, the pulp is living: and the appearance of the tooth and the surroundings indicate perfect health, and normal function: yet there is frequently experienced, some sharp pain, which she says is confined to the anterior portion of the root, evidently about half-way between the margin of the gum and the end of the root. There is no periostitus, no soreness of any kind. Passing an instrument between the gum and the tooth, I find no tarter or foreign matter of any kind. There is no pain, no soreness from percussion. As to treatment I have tried counter irritation, and indeed everything I could think of, but with no satisfactory result. The pain is not constant; but by steady pressure upon the gum immediately over the part affected, there is a slight soreness, but that is only when the pressure is made very strong. Now I have described the case as well as I can: please tell me what it is, and the treatment you would advise." C.

The case here detailed is evidently one of a neuralgic character induced by calcific deposit in the pulp, by which little granules, or nodules were formed: these are frequently the cause of true neuralgic pain. The question may occur, How do they occasion pain? By their location or size, they may be in contact with and impinge upon the nerve branches that ramify the pulp, and produce pain in the way that pressure upon nerve branches in any other part does. The mobility produced by the variation of the circulation, would doubtless cause impingement, and neural disturbance.

*Treatment.* Any local application upon the outside will afford no permanent relief. The patient should maintain the best possible general health; then the symptoms may abate, but it is then very uncertain. Systemic treatment should have in view one of two things, either the solution of the granule or the obtunding the sensibility of the nerve fibers. The accomplishment of the former, owing to the character, surroundings and peculiarities of the organ, would be wholly impracticable. Were it possible to give a solvent form to the fluids in the pulp to dissolve the granules in question, the vitality of the issue, would probably be destroyed; and even were that not the case, the walls of the chamber would be disolved as readily as the granules, and this would produce an abnormal condition that would be quite incompatible with true physiological construction and function.

The obtunding of the nerve branches would involve their death, or at least a diseased condition by which their proper function would be destroyed.

Two modes of treatment for such cases as the above, may be employed with certainty of success, at least so far as relief from pain is concerned. Extraction is effectual, but that involves the loss of the tooth, unless it is replaced.

The most practicable and efficient method is, to make a free and direct opening into the pulp chamber, and remove its contents and fill it with some proper material.

The pulp in such cases should be removed without using arsenious acid or any such material as a devitalizer. Local anæsthesia may be employed if the operation of removal would produce too much pain, or cause too much shock. Usually little or no local treatment is required before filling. In some cases there will be slight exudation for a time after the removal of a pulp; his should be abated before filling.

